# Simultaneous Real-Time Measurement of Isoprene and 2-Methyl-3-Buten-2-ol Emissions From Trees Using SIFT-MS

**DOI:** 10.3389/fpls.2020.578204

**Published:** 2020-11-27

**Authors:** Ann-Sophie Lehnert, Erica Perreca, Jonathan Gershenzon, Georg Pohnert, Susan E. Trumbore

**Affiliations:** ^1^Department of Biogeochemical Processes, Max Planck Institute for Biogeochemistry, Jena, Germany; ^2^Institute for Inorganic and Analytical Chemistry, Bioorganic Analytics, Friedrich Schiller University, Jena, Germany; ^3^Department of Biochemistry, Max Planck Institute for Chemical Ecology, Jena, Germany

**Keywords:** conifers, Picea, poplar, Pinus, VOC, isoprene, MBO, SIFT-MS

## Abstract

The C5 hemiterpenes isoprene and 2-methyl-3-buten-2-ol (MBO) are important biogenic volatiles emitted from terrestrial vegetation. Isoprene is emitted from many plant groups, especially trees such as *Populus*, while emission of MBO is restricted to certain North American conifers, including species of *Pinus*. MBO is also a pheromone emitted by several conifer bark beetles. Both isoprene and MBO have typically been measured by proton-transfer reaction mass spectrometry (PTR-MS), but this method cannot accurately distinguish between them because of their signal overlap. Our study developed a method for using selective ion flow tube mass spectrometry (SIFT-MS) that allows simultaneous on-line measurement of isoprene and MBO by employing different reagent ions. The use of *m*/*z*(NO^+^) = 68 u for isoprene and *m*/*z*(O_2_^+^) = 71 u for MBO gave minimal interference between the compounds. We tested the suitability of the method by measuring the emission of young trees of *Populus*, *Picea*, and *Pinus*. Our results largely confirm previous findings that *Populus nigra*, *Picea glauca*, and *Picea abies* emit isoprene and *Pinus ponderosa* emits MBO, but we also found MBO to be emitted by *Picea abies*. Thus SIFT-MS provides a reliable, easy to use, on-line measuring tool to distinguish between isoprene and MBO. The method should be of use to atmospheric chemists, tree physiologists and forest entomologists, among others.

## Introduction

The C5-hemiterpene isoprene, or 2-methyl-1,3-butadiene, is the most abundant biogenic volatile compound emitted from vegetation. Its annual global emission is estimated to be 350 to 769 Tg yr^–1^, approximately half of the total estimated emissions of biogenic volatile organic compounds (BVOC) (Guenther et al., 2012). Isoprene is emitted from mosses, ferns and higher plants, especially trees (Tingey et al., 1987; Hanson et al., 1999; Loreto, 2015). Angiosperms including species of *Populus* emit large amounts of isoprene, while in gymnosperms this hemiterpene is known to be emitted from species belonging to the genus *Picea*, including *Picea abies* and *Picea glauca*, but not from species belonging to the genus *Pinus*. Instead, the related hemiterpene 2-methyl-3-buten-2-ol (MBO), is emitted by *Pinus* species native to Northern America, e.g., *Pinus ponderosa*, *Pinus lodgepole*, and *Pinus jeffreyi* (Goldan et al., 1993; Harley et al., 1998). Globally, MBO contributions represent only a minor component of total BVOC emissions (Guenther et al., 2012), but in Northern American pine forests, their levels can reach 4–7 times the level of isoprene (Goldan et al., 1993; Harley et al., 1998; Schade and Goldstein, 2001).

2-Methyl-3-buten-2-ol and isoprene are often measured together as a sum parameter due to the experimental restrictions outlined below. However, it is important to distinguish between the two compounds during research in several different fields.

### Atmospheric Sciences

The oxidation of BVOCs, such as isoprene and MBO, in the atmosphere can produce tropospheric ozone in sufficiently NO-rich environments (Steiner et al., 2007), influencing air quality and, as ozone is a greenhouse gas, radiative warming. These compounds can also form secondary organic aerosols (Carlton et al., 2009), with both direct and indirect (as cloud condensation nuclei) impacts on radiative balance. Oxidation of MBO by OH-radicals represents one of the most important sources of acetone in those areas where it is emitted (Ferronato et al., 1998). However, the very different lifetimes of isoprene (2.8 h) and MBO (7 h) lead to different spatial and temporal distributions around areas of high emissions (Fantechi et al., 1998; Atkinson and Arey, 2003). Thus, the ability to measure these gases individually with a high time resolution would provide important insights into their relative roles in atmospheric chemistry and climate.

### Plant Sciences

Isoprene is thought to protect plants against abiotic stress by its antioxidant properties and stabilization of thylakoid membranes at high temperature (Perreca et al., 2020). Recently this molecule has also been proposed to activate gene networks involved in abiotic stress tolerance (Zuo et al., 2019). Although the role of MBO in plants has not been well studied, it is expected to be similar to that of isoprene based on a similar response of emission rates to light and temperature changes (Schade et al., 2000) and biosynthesis from the same substrate, dimethylallyl diphosphate (Gray et al., 2003). However, due to the differences in chemical properties, the way the two compounds serve in plant protection might differ. Especially the antioxidant properties of MBO might differ from those of isoprene. MBO was detected in the bark extracts of some angiosperms (Zhang et al., 2012) that are known to emit isoprene. Thus care should be taken to distinguish between the two compounds in simultaneous measurement in order to assess if their roles are different.

### Entomology

2-Methyl-3-buten-2-ol MBO is produced not only by trees, but also by tree pests. Several conifer bark beetles, e.g., the spruce bark beetle *Ips typographus*, produce MBO *de novo* as an aggregation pheromone (Bakke et al., 1977; Baader, 1989; Zhang et al., 2012). Thus simultaneous measurement of isoprene and MBO might allow for distinguishing between abiotic stress and bark beetle infestation in field measurements.

**FIGURE 1:**
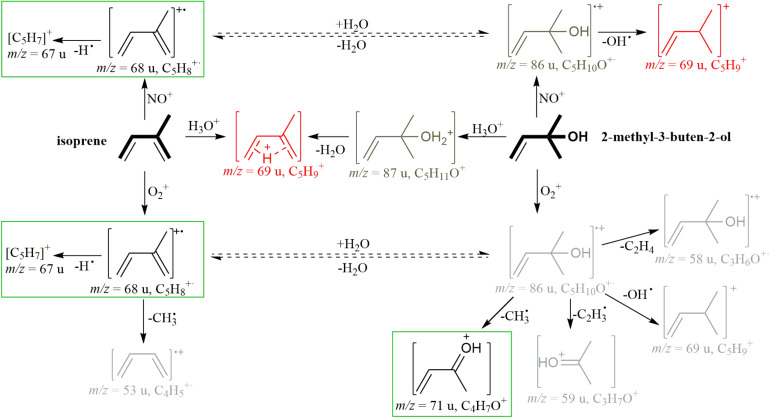
Scheme of ionization reactions of isoprene and MBO with the reagent ions H_3_O^+^, NO^+^, and O_2_^+^, based on Spanel and Smith (1998) and Schoon et al. (2007). Red ions are formed from both analytes, either by chemical reaction [*m*/*z*(H_3_O^+^) = 69 u] or by simple ionization of ^13^C isotopologues [*m*/*z*(NO^+^) = 69 u], and lead to overlaps in their spectra. Light gray ions are less intense than ions depicted in black. Ions marked by a green box were used to measure isoprene and MBO in this study. For legibility, additional ions that we found, but did not use for distinguishing the two compounds include *m*/*z* (H_3_O^+^: C_3_H_5_^+^ (41 u, both analytes), NO^+^: C_3_H_5_^+^ (41 u, MBO), C_4_H_5_^+^ (53 u, isoprene), O_2_^+^: C_2_H_3_O^+^ (43 u, MBO), were left out of the scheme. Dashed lines mark conversions that might be predicted to occur in the presence of water, but were found to play only a minor role based on experiments described in Figure 4 and in the text.

Proton-transfer reaction mass spectrometry (PTR-MS) and gas chromatography-mass spectrometry (GC-MS) are the most-widely used techniques for measuring BVOCs. PTR-MS ionizes gaseous analytes with H_3_O^+^ ions in a drift tube with a defined reaction time and detects them via mass spectrometry with a quadrupole or Time of Flight mass analyzer. This technique allows for online-measurements of gaseous analytes at low mixing ratios (Krechmer et al., 2018). However, with PTR-MS, the [M+H-H_2_O]^+^ fragment of MBO and the [M+H]^+^ signal of isoprene have the same mass to charge ratio (cf. Figure 1, H_3_O^+^ pathway), so accurate distinction of the two compounds is difficult. GC-MS allows for a separation of the analytes based on differences in retention time and mass spectra, but is not suitable for online monitoring due to relatively long measurement times. In addition, GC-MS often requires pre-concentration on, e.g., cartridges before measurement. In practice, both techniques are usually employed side by side, using PTR-MS to acquire good time resolution and GC-MS for identification (Jardine et al., 2020). Another approach involves switchable reagent ion mass spectrometry (SRI-MS), a technique similar to PTR-MS, but including additional ion sources for NO^+^, O_2_^+^, NH_4_^+^, Kr^+^, and Xe^+^ (Jordan et al., 2009). Using the NO^+^ ion, isoprene can be detected at *m*/*z*(NO^+^) = 68 u, and MBO at *m*/*z*(NO^+^) = 69 u, as has been shown in a field study with SRI-MS (Karl et al., 2012), but these instruments are very costly and complex to operate. Because of the important role that isoprene and MBO play in different scientific fields, the possibility to distinguish between these compounds with accuracy using online measurements is desirable, but until now not realized technically with readily available instruments.

Selected ion flow tube mass spectrometry (SIFT-MS) is a cheaper (though still rather costly) and easy-to-use alternative to SRI-MS. Like PTR-MS and SRI-MS, SIFT-MS is a chemical ionization mass spectrometry technique for measuring gaseous analytes. Unlike PTR-MS, it utilizes multiple reagent ions that react differently with gaseous analytes, so one can obtain more structural information from the respective spectra. By measuring one analyte with more than one reagent ion, multiple spectra are generated. Comparison of these spectra allows identification of ions with the least interference from other VOCs and thus specific quantification of the target analyte.

In SIFT-MS, reagent ions are generated by a moist air plasma and then selected by a quadrupole. Reagent ions and gaseous analytes are mixed in a flow tube that is flushed continuously with a carrier gas. They travel together, and their reaction time is determined by the time they need to cross the flow tube (Smith and Spanel, 2011). During this time, the analytes are ionized during collision with the reagent ions. In our case, the reagent ions used were H_3_O^+^, NO^+^, and O_2_^+^, but it is also possible to use the negative ions OH^–^, O^–^, O_2_^–^, NO_2_^–^, and NO_3_^–^. With H_3_O^+^, mostly proton transfer reactions occur, with NO^+^ electrons are transferred or NO^+^ adducts are formed and with O_2_^+^, electrons are transferred and sometimes fragmentation reactions occur. The product ions and remaining reagent ions are detected via a quadrupole mass analyzer. A library is implemented in the software of the manufacturer that can be used to calculate their mixing ratio directly from the measured counts.

When measuring isoprene and MBO with SIFT-MS, H_3_O^+^ generates *m*/*z*(H_3_O^+^) = 69 u for both analytes, like in PTR-MS (cf. Figure 1 for a reaction scheme). However, with NO^+^, isoprene generates *m*/*z*(NO^+^) = 68 u, and MBO generates *m*/*z*(O_2_^+^) = 69 u. With this difference, one can measure isoprene well, but the ^13^C isotopologue of isoprene interferes with MBO measurement. With O_2_^+^, isoprene forms two product ions, *m*/*z*(O_2_^+^) = 67 u and 68 u, at similar intensities, whereas MBO mostly forms *m*/*z*(O_2_^+^) = 71 u (Spanel and Smith, 1998; Schoon et al., 2007).

In this study, we demonstrate the use of SIFT-MS for simultaneous measurement of isoprene and MBO by monitoring isoprene with *m*/*z*(NO^+^) = 68 u and MBO with *m*/*z*(O_2_^+^) = 71 u. To validate our method, we performed measurements on three different isoprene-emitting tree species, *Populus nigra*, *Picea abies*, and *Picea glauca*, and on *Pinus ponderosa*, which is reported to emit MBO.

## Materials and Methods

### Supplies and General Remarks

Isoprene, MBO, and dodecane were purchased from Sigma Aldrich (Darmstadt, Germany). Deuterated water was purchased from TCI (Eschborn, Germany). Distilled water was generated by a Enviro FALK GEO + EDI 200 electrode ionization cell (Enviro FALK, Westerburg). The tubing used for the tree chamber experiment was opaque black 1/4” PFA-tubing, the tubing used for the standard measurements was opaque black1/8” PFA-tubing. Connectors for the tree experiment were Galtek PFA fittings (Entegris, United States), for the standard measurements and calibrations Swagelok stainless steel fittings (Swagelok, United States). All setups were built such that an overflow line to room air ensured ambient pressure in the chamber and at the SIFT-MS inlet.

### SIFT-MS Settings

Measurements were conducted with a Voice 300 ultra SIFT-MS (Syft Technologies Ltd., Christchurch, New Zealand) with a positive ion source that was customized as described by Lehnert et al. (2019). 40 cm^3^/min sample gas flow, 156 cm^3^/min helium carrier gas flow, 50 V flow tube voltage, 120°C flow tube temperature, and 105°C sample plate and sampling line heater temperature were used. To suppress dimer formation at high mixing ratios, the larger trees (*P. nigra*, *P. abies*, and *P. glauca* #1) were measured at 390 cm^3^/min carrier gas flow.

The ratio of the reagent ions NO^+^ and O_2_^+^ varied between the different experiments. However, this did not affect our results significantly since for the interference calculation, measurements from the same ions were used, and for the calculation of mixing ratios and release rates, the ratios of product ion to reagent ion were used.

### SIFT-MS Measurements of Isoprene and MBO Standards

Full mass spectra were measured for both standards using a diffusion cell flushed with VOC-free air from a pure air generator (PAG 003, Ecophysics, Dürnten, Switzerland). An 1.5 mL vial with Teflon septum was filled with 50 μL isoprene or MBO. A thin needle (23 G × 1”) was pierced through the septum, and then the vial was placed in a 40 mL headspace vial that was flushed with 0.5 L/min pure air humidified to 0, 25%, 50%, 75%, and 100% relative humidity at 25°C by a GCU gas calibration unit (IONICON Analytik GmbH, Innsbruck, Austria). The method captured ion counts between 10 and 250 u for all three reagent ions. The dwell time was 100 ms, and the count limit 10.000. 10 scans of each substance were measured and averaged.

For distinguishing the two standards, a selected ion monitoring (SIM) scan was set up for 10 min, with 500 ms dwell time/scan time and 100.000 cps count limit (36 scans, first and last omitted for averaging). The masses used are listed in Supplementary Table S1. The increased count limit and scan time compared to standard SIFT-MS settings was used to decrease variability in the measured reagent ion counts. The maximum ratio of product to reagent ions was 3%, so the assumption that the reagent ion counts remain unchanged in the flow tube is still valid.

### Ionization of Isoprene and MBO in the Presence of Deuterated Water

We humidified pure air by bubbling it through deuterated water at room temperature. This moist air was mixed with pure air that was enriched in isoprene or MBO, respectively, by passing it over water with 1 μL isoprene or MBO in the diffusion cell. Both flows were 400 mL/min. Mass spectra were recorded between *m*/*z* = 15 and 150 u, with a scan time limit 1 s, count limit 100.000 counts, and four repeats per measurement. As a control, the experiment was repeated with normal, non-deuterated water.

### Tree Cultivation

*Populus nigra* trees were grown from stem cuttings obtained from trees grown in a common garden of *P. nigra* accessions in Isserstedt, Germany. The 1-year-old trees were grown in the greenhouse of the Max Planck Institute of Chemical Ecology (MPICÖ) Jena, Germany under the following conditions: 20/18°C (day/night), relative humidity 60%, natural light with 9–14 h photoperiod, and supplemental light for 12 h, with SON-T Agro lamps (Philips, Andover, MA, United States).

Three-year-old *Picea abies* trees were planted originally from seeds in 2016 and were grown outdoors in the garden of the MPICÖ, until the experiment was performed. Trees were irrigated every day. One-year-old *Pinus ponderosa* trees were obtained from a local nursery in Thuringia. Four-year-old *Picea glauca* trees (accession #1) were obtained from a local nursery in Thuringia in 2017 and grown prior to the experiment outdoors in the garden of the MPICÖ. Trees were irrigated every day. Three-year-old *Picea glauca* (accession #2) trees were obtained as seedlings from the Laurentian Forestry Centre, Quebec, Canada, in 2016, and grown under controlled environmental conditions in a growing chamber in the MPICÖ until the start of the experiment. Summer (16/8 h for day/night, 22°C and photosynthetically active radiation (PAR) 1000 μmol/m^2^/s) and winter (8/12 h for day/night, 5°C and PAR 200 μmol/m^2^/s) conditions were alternated for 6 months (summer) and 3 months (winter) in the chamber.

### Isoprene and MBO Emissions From Trees

Prior to the experiment, the trees were moved to the greenhouse of the Max Planck Institute of Biogeochemistry, Jena, Germany, and kept there for 4 weeks. The greenhouse was set up at 60% humidity and a 12 h day/night cycle (30°C/25°C). LED-lights (ultra violet, <400 nm, 1%; blue, 400–500 nm, 20%; green, 500–600 nm, 39%; red, 600–700 nm, 35%; far-red, 700–800 nm, 5%; Valoya, Finland) illuminated the trees with a PAR of 150 μmol/m^2^/s and were supplemented by ambient light entering the greenhouse, reaching a PAR level of 300–400 μmol/m^2^/s. Trees were watered daily. Before performing the measurement of isoprene and MBO with the SIFT-MS, trees were put into the chamber for 24 h [setup similar to Huang et al. (2018), scheme in Supplementary Figure S1].

The three tree-containing cylindrical chambers plus one reference chamber without a tree were made from FEP-foil. These chambers (height = 50 cm, diameter = 40 cm, volume = 60 L) were mounted in a polyacrylate scaffold. A Teflon tube ring with holes was placed at the bottom of the chambers and connected to an air inlet. Compressed air was dried and purified on adsorber columns, after which CO_2_ was added back in to achieve levels of 400 ppm. Rotameters regulated the air flow through the chamber to 3 L/min. 1/4” black PFA tubing of 2 m length connected the chamber to the instrument. The outlets of the VICI-valve on the SIFT-MS were connected via T-pieces and 1 m tubing to a pump flushing the tubes from the chambers to the instrument at all times. Photosynthetically active radiation (PAR) and temperature were tracked in each chamber. Tree emissions were measured via the SIM scan described above for 24 h capturing a full diurnal cycle. After measuring each chamber with the SIM scan described above, additionally, one full mass spectrum was also measured for every chamber plus the instrument’s internal background with the settings mentioned above with a single scan.

Mixing ratios were calculated as:

(1) $$\begin{array}{c} \chi_{isoprene}=1.0035\cdot 10^{-10}\cdot \frac{T_{FT}}{p_{FT}}\cdot \left(\frac{\varphi_{carr}}{\varphi_{samp}}+1\right)\cdot \\ \frac{I\left(NO^{+},68u\right)\cdot ICF\left(NO^{+},68u\right)}{\begin{array}{c} k_{isoprene,NO^{+}}\cdot br_{isoprene,NO^{+},68u}\cdot \\ I\left(NO^{+},30u\right)\cdot ICF\left(NO^{+},30u\right) \end{array}} \end{array}$$

and

(2) $$\begin{array}{c} \chi_{MBO}=1.0035\cdot 10^{-10}\cdot \frac{T_{FT}}{p_{FT}}\cdot \left(\frac{\varphi_{carr}}{\varphi_{samp}}+1\right)\cdot \\ \frac{I\left(0_{2}^{+},71u\right)\cdot ICF\left(O_{2}^{+},71u\right)}{\begin{array}{c} k_{MBO,O_{2}^{+}}\cdot br_{MBO,O_{2}^{+},71u}\cdot I\left(O_{2}^{+},32u\right)\cdot \\ ICF\left(O_{2}^{+},32u\right) \end{array}}, \end{array}$$

χ is the mixing ratio in ppb, *T*_FT_ the flow tube temperature in K, *p*_FT_ the flow tube pressure in mTorr, φ_carr_ the carrier gas flow in Torr L/s, and φ_samp_the sample gas flow in Torr L/s. *I*() is the intensity of the ion ionized by the reagent ion and measured at the mass stated, *ICF*() the instrument calibration factor at the ion as specified, *k* the kinetic rate constant in cm^3^⋅molecule/s of the reaction with isoprene/MBO with the respective reagent ion, and *br* the branching ratio of the measured ion. The branching ratios were determined from the standard measurements at 100% humidity, which had the most similar 19/37 signal to the samples. The mixing ratios were calculated for each scan omitting the first and last scan of each measurement, and then the mean and standard deviation were calculated from this. The maximum observed ratio of product ion to reagent ion was 0.9% in the case of isoprene emissions from poplar, which is low enough to fulfill the assumption that the reagent ion counts did not change significantly. The maximum ion counts of NO^+^⋅H_2_O, an additional reagent ion water cluster, were always below 500 cps, which corresponded to less than 0.25% of the reagent ion counts and was thus not included in the calculation.

From there, the emission rate was calculated as

(3) $$\phi =\frac{\chi \cdot M\cdot \varphi_{air}}{V_{mol}\cdot m_{leaves/needles,dry}}$$

ϕ is the release rate in μmol/(g h), *M* is the molar mass of the compound, φ_air_ is the air flow through the incubation chamber (cf. Supplementary Table S3), *V*_mol_ is the molar volume, used 24 L/mol as it was 25°C in the chamber, and *m*_leaves/needles,      dry_ is the leaf or needle dry mass (cf. Supplementary Table S2).

The error of the emission rate was calculated as

(4) $$\begin{array}{c} \text{Δ}\phi =\frac{t\left(95\%,n_{meas}-1\right)}{n_{meas}-1}\cdot \\ \sqrt{\begin{array}{c} M\cdot ({\left(\frac{\phi_{air}\cdot \text{Δ}\chi }{m_{leaves/needles,dry}}\right)}^{2}+{\left(\frac{\chi \cdot \varphi_{air}\cdot \text{Δ}V_{mol}}{V_{mol}^{2}\cdot m_{leaves/needles,dry}}\right)}^{2}+\\ {\left(\frac{\chi \cdot \text{Δ}\varphi_{air}}{V_{mol}\cdot m_{leaves/needles,dry}}\right)}^{2}+{\left(\frac{\chi \cdot \varphi_{air}\cdot \text{Δ}m_{leaves/needles,dry}}{V_{mol}\cdot m_{leaves/needles,dry}^{2}}\right)}^{2}) \end{array}} \end{array}$$

with *t*(95%, *n*_meas_−1) the result of the t-distribution at 95%. The degrees of freedom are the number of measurements per time point – 1 (21–22, depending on the measurement). Δχ is the standard deviation of the mixing ratios that was calculated based on the 21–22 individual measurements per time point. Δ*V*_mol_ = 0.72 L/mol is the error of the molar volume for 5 K and 0.02 bar deviation of the temperature and pressure. Δφ_air_ is the reading error of the gas flow measurements, and Δ*m*_leaves/needles, dry_ the reading error of the weight measurement.

## Results

**FIGURE 2:**
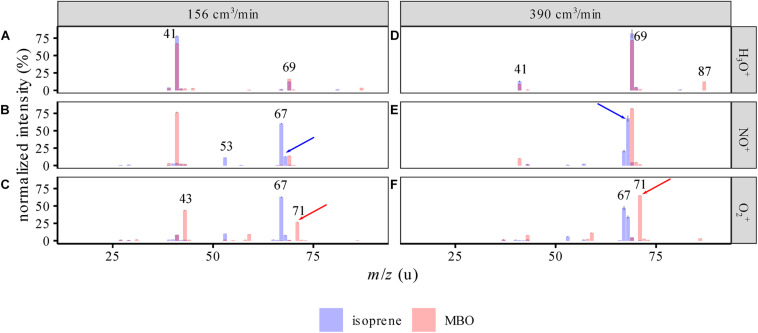
SIFT-MS spectra of isoprene and MBO standards for the different reagent ions (mean ± 95% CI, *n* = 9), at 100% humidity at 25°C. **(A–C)** Spectra with 156 cm^3^/min carrier gas flow, **(D–F)** spectra with 390 cm^3^/min carrier gas flow. The intensities are normalized to the largest peak in the area of the spectrum depicted. Both spectra are superimposed and semitransparent, so purple means both intensities coincide at this *m/z*. For example, in the top panel *m/z* = 69, which represents the main signal used for isoprene in PTR-MS measurements, here overlaps with an MBO signal and also generates a fragment ion which is specific for neither isoprene nor MBO. Red and blue arrows identify the ions used to measure isoprene and MBO in this study. The numbers show the *m/z* of high intensity peaks.

Mass spectra of the standards (Figure 2) showed that isoprene and MBO react differently with NO^+^ and O_2_^+^ than previously described (Spanel and Smith, 1998; Schoon et al., 2007). The biggest difference lies in the finding of a strong signal for isoprene at *m/z* = 67 u upon reaction with NO^+^. As the carrier gas flow used and moisture level were similar to those in our study (390 ccm at 100% humidity), we attribute the spectral changes to an increased ion energy leading to increased fragmentation due to the higher flow tube voltage and temperature settings. We observed a decrease in fragmentation when increasing the carrier gas flow (Figures 2A–C vs. D–F), probably a result of product ions transferring excess energy more rapidly to the carrier gas due to an increased number of collisions.

**FIGURE 3:**
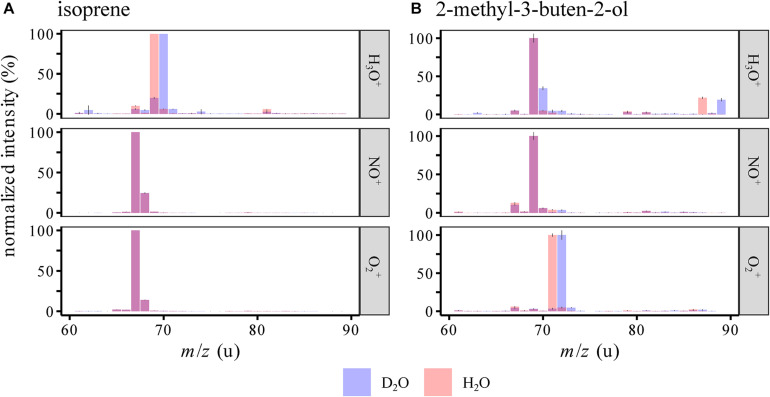
Interference of the isoprene signal on the MBO masses *m*/*z*(NO^+^) = 69 u and *m*/*z*(O_2_^+^) = 71 u normalized to the isoprene signals at *m*/*z*(NO^+^) = 68 u and *m*/*z*(O_2_^+^) = 67 u **(A)**, and interference of the MBO signals on the isoprene masses *m*/*z*(NO^+^) = 68 u and *m*/*z*(O_2_^+^) = 67 u, normalized to the MBO signals at *m*/*z*(NO+) = 69 u and *m*/*z*(O_2_^+^) = 71 u **(B)**, at different relative humidities. Original reagent ion counts were 2**−**3E6 cps, and original product ion counts 1.0**−**1.5E5 cps. For all interferences, values were estimated for 156 cm**^3^**/min and 390 cm**^3^**/min carrier gas flow. As in PTR-MS, an increased humidity can increase back reactions (**[*M*−*H*]^+^ + *H*_2_*O*→*M* + *H*_3_*O*^+^**), water cluster formation (**[*M*]^+^ + *H*_2_*O*→[*M*⋅*H*_2_*O*]^+^**), and other reactions involving water, leading to changes in product ion counts for the same measured analyte concentration with changing humidity. An increase in the carrier gas flow leads to more collision partners that can take up energy, so thermodynamically stable products are favored over kinetic products. We only used *m*/*z*(O_2_^+^) = 71 u for measuring MBO and *m/z*(NO^+^) = 68 u for measuring isoprene, as the other interferences were much higher with a higher uncertainty.

Upon reaction with NO^+^, one could potentially use the *m*/*z* = 67 or 68 u signals for measuring isoprene and the *m*/*z* = 69 u for measuring MBO, as described by Karl et al. (2012). However, limitations in the mass resolution of the quadrupole used in the SIFT-MS resulted in a 5–6% interference [normalized to *m*/*z*(NO^+^) = 68 u] due to the natural isotopologues of isoprene substituted with a single ^13^C. When we measured the isoprene standard at different carrier gas flows and humidities, we determined 6% to 8% interference of isoprene with the MBO signal at *m*/*z*(NO^+^) = 69 u (Figure 3). Corrected for the ^13^C isotope peak, this calculates to a secondary reaction of isoprene to C_5_H_9_^+^ (*m*/*z* = 69 u) with 1–2% abundance. An explanation for the formation of this ion could be H_2_O addition and subsequent OH^⋅^ loss, cf. Figure 1. Using *m*/*z*(O_2_^+^) = 71 u for measuring MBO was more accurate than using *m*/*z*(NO^+^) = 69 u, as the interference of isoprene at *m*/*z*(O_2_^+^) = 71 u was below 1% of that at *m*/*z*(O_2_^+^) = 67 u.

We also measured up to 0.7% interference of MBO on the isoprene signal at *m*/*z*(NO^+^) = 68 u, normalized to *m*/*z*(NO^+^) = 69 u. This interference was more prominent in dry samples since MBO ionized by NO^+^ [C_5_H_10_O^+^, *m*/*z*(NO^+^) = 86 u] might not only eliminate OH radicals to form C_5_H_9_^+^ [*m*/*z*(NO^+^) = 69 u], but also eliminate water to form C_5_H_8_^+^ [*m*/*z*(NO^+^) = 68 u]. In this case, the presence of water would make this side reaction less favorable due to the principle of Le Chatelier. On the other hand, if we had used *m*/*z*(O_2_^+^, 67 u) to measure isoprene, the interference and thus the error would have been much higher, up to 1.9%.

To distinguish between isoprene and MBO, we sought signals of each compound that had the least interference from the other compound to minimize error, which scales with signal intensity. Thus, since the interference of isoprene on *m*/*z*(O_2_^+^, 71 u) is much smaller than on *m*/*z*(NO^+^) = 69 u, we chose the former for measuring MBO. And, since the interference of MBO on *m*/*z*(NO^+^) = 68 u is smaller than on *m*/*z*(O_2_^+^, 67 u), we chose the former for measuring isoprene. If the differences in mixing ratios between the two compounds are not anticipated to be large, and rapid measurements are needed with just a single reagent ion, it would be best to use both O_2_^+^ ions, *m*/*z*(O_2_^+^, 67 and 71 u), as the interference is lower than for the two NO^+^ ions and one saves the time of measuring both reagent ions.

**FIGURE 4:**
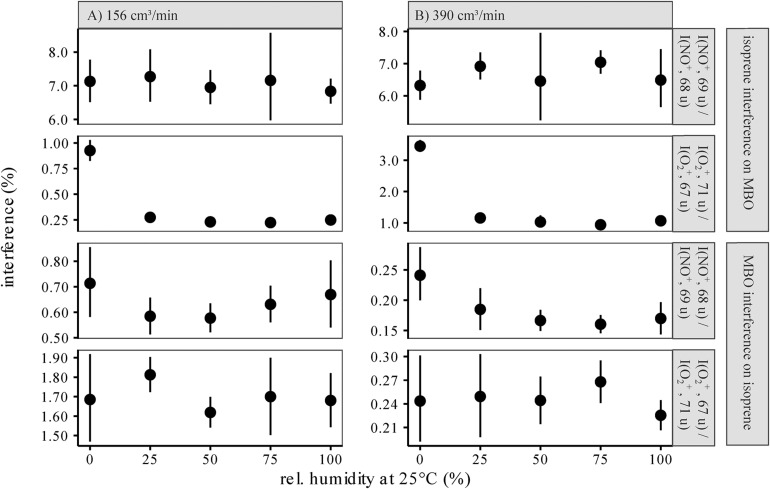
SIFT-MS spectra of isoprene and MBO in humid air (mean ± 95% CI), humidified by either normal or deuterated water for both 156 cm^3^/min **(A)** and 390 cm^3^/min **(B)** carrier gas flows. Intensities were normalized to the largest peak in the area of the spectrum depicted. Evidence for the involvement of water in flow tube reactions comes from the shifts *m*/*z*(H_3_O^+^) = 69→70 u for isoprene and MBO as well as *m*/*z*(H_3_O^+^) = 85→87 u and *m*/*z*(O_2_^+^) = 71→72 u for MBO when D_2_O is present instead of H_2_O. Interestingly, for isoprene ionized by NO^+^ and O_2_^+^, no changes were observed, so the reactions are apparently affected by water but do not involve a hydrogen atom that can be exchanged for deuterium.

2-Methyl-3-buten-2-ol is formally an isoprene molecule with the addition of water to the substituted double bond. It could thus be possible that an ionized form of isoprene could react to form MBO in the presence of water. For example, isoprene ionized by NO^+^ forms C_5_H_8_^+^, which could react with water to form C_5_H_10_O^+^ with the same structure and *m*/*z* as ionized MBO. To evaluate the role of water in these proposed flow tube reactions, the standards were measured in air humidified by either H_2_O or D_2_O (Figure 4). If water is involved in the reaction, and a deuterium from water is added to or exchanged with the ion, the measured mass would be 1 u higher due to the higher mass of deuterium compared to hydrogen. The reagent ions (see Supplementary Figure S2) showed an influence only in the H_3_O^+^ channel, where H_2_DO^+^, HD_2_O^+^, and D_3_O^+^ are detected. As expected, we saw a mass shift from *m*/*z*(H_3_O^+^) = 69 u to 70 u for both isoprene and MBO, as the reagent ions were both saturated and thus the protonation added a D to the analytes. *m*/*z*(O_2_^+^) = 71 u shifted to 72 u for MBO, so here, also water vapor was involved in forming this ion. Interestingly, for isoprene, the NO^+^ and the O_2_^+^ signals did not change at all, so no proton exchange occurred in the formation of isoprene ions. This contradicts the hypothesis that water is involved in forming *m*/*z*(NO^+^) = 69 u and *m*/*z*(O_2_^+^) = 71 u. However, we did observe an increase in the relative abundance of those two peaks when switching from dry to wet sample air. This could be due to suppression of the isoprene ions at *m*/*z*(NO^+^) = 68 u and *m*/*z*(O_2_^+^) = 67 u by excess water.

**FIGURE 5:**
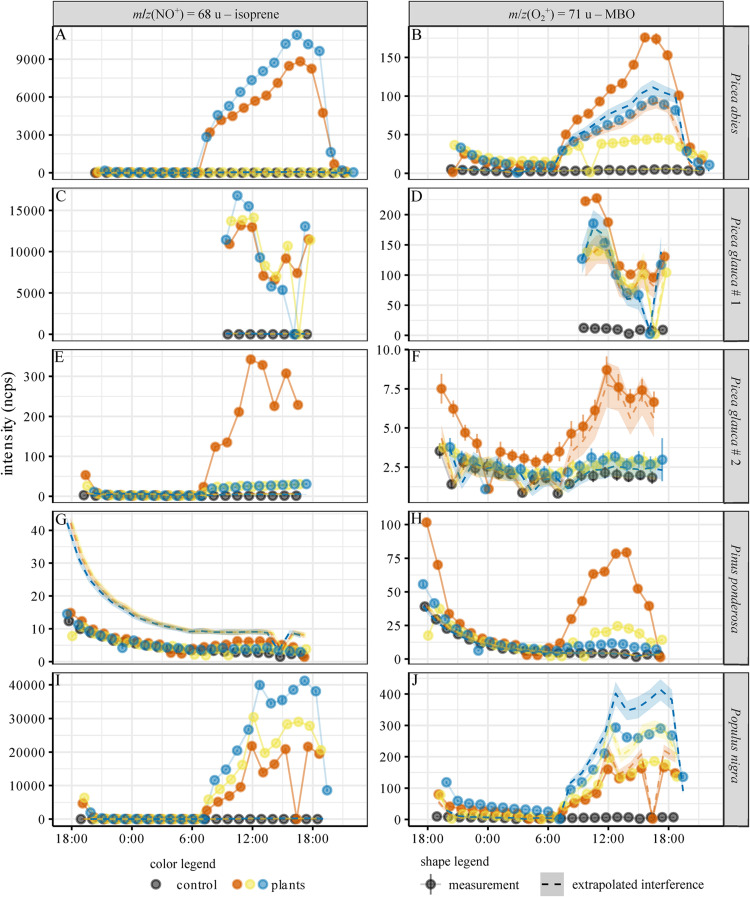
Diurnal cycle of isoprene and MBO emissions for the tree species investigated. **(A,C,E,G,I)**: Intensity of isoprene proxy, **(B,D,F,H,J)**: Intensity of MBO proxy. Emissions of Picea abies **(A–B)**, Picea glauca #1 **(C–D)** and #2 **(E–F)**, Pinus ponserosa **(G–H)** and Populus nigra **(J)**. The measured intensities were normalized to 10^6^ reagent ion counts. Black: control, empty chamber. Different colors indicate the measurements for the three replicate trees used for each species. Dots: mean ± 95% CI of the SIFT-MS measurement. A sudden zero value indicates instrument malfunctioning (before a firmware update, software did not always switch on the VICI valve for long measurements). Dashed lines: interference extrapolated from *m*/*z*(NO^+^) = 69 u (MBO interference on the isoprene signal) and *m*/*z*(O_2_^+^) = 67 u (isoprene interference on the MBO signal) – mean ± 95% CI. Basically, if the signal at *m*/*z*(O_2_^+^) = 67 u is isoprene, then a maximum of 1.4% (for lower carrier gas flows) and 2.9% (for higher carrier gas flows) of this signal will be seen at *m*/*z*(O_2_^+^) = 71 u where we measure MBO. These values are represented by the dashed lines in the graphs. If the intensity of *m*/*z*(O_2_^+^) = 71 u is higher than this signal, this is taken as evidence for the presence of genuine MBO. This also works the other way around, for MBO interference on isoprene.

To test our ability to distinguish between isoprene and MBO in an experimental setup with natural sources of these gases, emissions from *Picea glauca*, *Picea abies*, *Populus nigra*, and *Pinus ponderosa* were measured over the course of a day. All trees exhibited a diurnal cycle of BVOC emissions related to the presence of light (Figure 5). Isoprene was measured from *m*/*z*(NO^+^) = 68 u, and MBO was measured from *m*/*z*(O_2_^+^) = 71 u. We calculated the contribution of isoprene to the *m*/*z*(O_2_^+^) = 71 u signal with the equation $I\left(O_{2}^{+},71u,isopreneinterference\right)=x\cdot I\left(O_{2}^{+},67u,isoprene\right)$ (Figure 3). If the measured MBO signal is equal or below this value, the signal is not significantly different from the expected isoprene interference and no MBO is actually detected. If the signal is higher, it is measured as MBO. This of course also applies also for the MBO interference on isoprene, with *I*(*NO*^+^, 68*u*,*MBOinterference*) = *x*⋅*I*(*NO*^+^, 69*u*,*MBO*). Again, we used the interference ratio that was determined measuring the standards at 100% humidity, as the trees transpired a substantial amount of water, leading to high air humidity in the chambers. A visualization of the potential isoprene interference with MBO for each species can be found in Supplementary Figure S4, where we plotted I(O_2_^+^, 67 u) against I(O_2_^+^, 71 u). Each species shows linear dependence of the two signals, but only *Picea abies* and *Pinus ponderosa* are found significantly above the black line, and so must emit MBO.

Relative humidity does not affect the ability to distinguish between isoprene and MBO except under very dry conditions (Figure 3). However, we suggest that interference rates should be determined with standards under conditions as close to the experiment as possible to rule out possible errors.

For each tree species, we analyzed the emission of isoprene and MBO from three individual trees, represented in Figure 5 by different colors (blue, red, and yellow). The extrapolated interference signals are indicated with a dashed line in the color used for each individual tree. All species except *P. ponderosa* showed isoprene emissions (Figure 5), and within the tree species, the emission intensity mostly correlated with the needle or leaf dry mass, cf. Supplementary Table S2. For *P. abies*, the isoprene signal for one tree (shown in yellow) was a factor of 150 smaller than the other two (Figure 5A and Supplementary Figure S3), even though the biomass of this tree individual was the highest (Supplementary Table S2). For *P. glauca* (accession #2), one tree (red) had an isoprene signal 10 times higher than the other two, even though the biomass was comparable (Figure 5E and Supplementary Table S2). Intraspecific variation was smaller among *P. glauca* (accession #1) and *P. nigra* (Figures 5C,I). As the isoprene signal of *m*/*z*(NO^+^) = 68 u was much higher than the MBO signal of *m*/*z*(NO^+^) = 69 u, and the calculated interference of MBO based on *m*/*z*(NO^+^) = 69 u was close to the control in all cases, the isoprene signal did not result from interference of MBO. The isoprene signal for *P. ponderosa* trees was lower than the interference signal extrapolated from the MBO emissions on *m*/*z*(NO^+^) = 69 u (Figure 5G), so we conclude that, as expected, these trees did not emit isoprene, and that the signal at *m*/*z*(NO^+^) = 68 u in these cases arose from MBO.

A strong signal at *m*/*z*(O_2_^+^) = 71 u was observed from the emissions of *P. ponderosa*. It can be assigned to MBO since no isoprene emission was observed from this plant and thus no interference of this analyte has to be considered. The signals at *m*/*z*(O_2_^+^) = 71 u of both *P. glauca* accessions and *P. nigra* can be attributed to isoprene based on the isoprene signal [*m*/*z*(O_2_^+^) = 67 u]. These trees thus do not produce MBO. Interestingly, two of the *P. abies* individuals emitted MBO as well (Figure 5B). The ratio of isoprene and MBO signals differed substantially between the individual trees. Trees indicated with red and blue in Figure 5 show much higher isoprene emissions at *m*/*z*(NO^+^) = 68 u than the tree labeled with yellow. In contrast, the MBO emissions at *m*/*z*(O_2_^+^) = 71 u of the three trees are in the same intensity range. Possible interference by other terpenes was considered improbable since no other naturally occurring hemiterpenes are known, and monoterpene emissions measured at *m*/*z*(H_3_O^+^) = 137 u, *m*/*z*(NO^+^) = 136 u, and *m*/*z*(O_2_^+^) = 136 u were a factor of 10–100 lower than the measured isoprene and MBO intensities. Only fragment ions from monoterpenes would overlap with isoprene and MBO, and their branching ratio should decrease the intensity even further.

Based on the measured intensities and eq. (1)–(4) above, we calculated the release rates of isoprene and MBO for mid-day (noon), cf. Table 1 and Supplementary Figure S4. Given the low standard deviation, our results give a good idea of relative emission rates for the tree species and individuals involved. Since we could not calibrate the measured intensities as our VOC standard was not concentrated enough to capture the mixing ratio range of the plants, we had to rely on the internal instrument calibration described by Smith and Spanel (2005). As the uncertainty of measurements without external calibration is estimated to be at least ±35% (Langford et al., 2014) including systematic error, our results may not be very accurate. Calculating the isoprene emission rates based on *m*/*z*(O_2_^+^) = 67 u led to fluxes about 2/3 to 1/2 as high as the fluxes calculated from *m*/*z*(NO^+^) = 68 u (Supplementary Figure S6). Thus when rigorous quantification is needed, we strongly recommend calibration.

**TABLE 1:** Isoprene and MBO emission rates in μg/(g_*dry weight*_ × h) at 12:00 noon.

	Isoprene			MBO		
	1	2	3	1	2	3
*P. abies*	8.8 ± 0.2	0.040 ± 0.002	19.5 ± 0.4	0.125 ± 0.006	0.029 ± 0.002	n.s.
*P. glauca 1*	41.7 ± 0.8	13.3 ± 0.2	64 ± 1	n.s.	n.s.	n.s.
*P. glauca 2*	25.2 ± 0.7	2.9 ± 0.2	3.3 ± 0.2	n.s.	n.s.	n.s.
*P. nigra*	175 ± 7	174 ± 4	126 ± 3	n.s.	n.s.	n.s.
*P. ponderosa*	n.s.	n.s.	n.s.	1.42 ± 0.05	0.79 ± 0.05	0.95 ± 0.05

*Values listed represent the mean ± 95% CI. n.s.: After correcting for interference of the other analyte, the signal was not significantly different from 0 (p = 95%). The confidence intervals are calculated based on the measured standard deviations of the intensities, however, these values can only be considered as estimates of the emissions, as the instrument is reported to have an actual error of ±34% (Langford et al., 2014) for the mixing ratio calculation employed.*

## Discussion

Using SIFT-MS, we developed a method that allows distinguishing between isoprene and MBO in online measurements. For scientific questions where monitoring of both compounds is essential, e.g., for investigation of drought stress and bark beetle infestation or for monitoring BVOC emissions at the ecosystem level, this is a reliable, easy method. Full scans of isoprene and MBO analytical standards allowed the selection of the ions *m*/*z*(NO^+^) = 68 u for measuring isoprene and *m*/*z*(O_2_^+^) = 71 u for measuring MBO. These intense ions show the least interference with signals from the other compound and allow a stable and reliable online measurement of the analytes. As proof of concept we applied the method to the determination of isoprene and MBO emissions during the diurnal cycle in five tree species.

SIFT-MS is capable of measuring isoprene and MBO simultaneously under most conditions because of minimal interference between the two compounds for the diagnostic signals we have selected. However, the ratio of these signals depends on the operating conditions of the instrument, especially sample humidity. Thus, these ratios should be determined with standards under identical measurement conditions as used for the sample.

For more accurate quantification of small amounts of isoprene or MBO in the presence of large amounts of the other compound, one could include the humidity-dependence in the interference calculation. In $I\left(O_{2}^{+},71u,isopreneinterference\right)=x\cdot I\left(O_{2}^{+},67u,isoprene\right)$, *x* could be replaced by a term dependent on the sample humidity, e.g., $x=a\cdot I\left(O_{2}^{+},19u\right)+b$, which requires a humidity-dependent calibration of all ions. For simplicity, we decided to use the interference factors determined at very high humidity, as this was closest to the humidity in our experiment.

With SIFT-MS, isoprene and MBO can be determined in a single run. In previous approaches described for this analytical problem, a rather laborious measurement of the analytes with GC-MS for identification and PTR-MS for quantification was employed (Jardine et al., 2020). Using PTR-MS, complex calculations were required for a semiquantitative determination of the analytes. The SIFT-MS method introduced here represents a substantial simplification. With the Eqs (1) and (2), no tedious, humidity-dependent calibration is necessary as for PTR-MS. For increased accuracy, a calibration is advised for SIFT-MS as well (Langford et al., 2014; Lehnert et al., 2019).

Isoprene can even be determined in a 50-fold excess of MBO with SIFT-MS, as the MBO interference signal on the isoprene signal is only 0.5%. MBO determination can be accomplished in the presence of a 20-fold excess of isoprene. Limitations to the method are only to be expected if other analytes with the same mass to charge ratios as used for quantification of isoprene and MBO are present in the VOC mixture of the samples. Isoprene and MBO are abundant in natural BVOC samples (Penuelas and Staudt, 2010), thus this limitation should rarely be a major problem.

The ionization mechanism of the two structurally related analytes was investigated by using deuterated water for air humidification. If *m*/*z*(NO^+^) = 69 u could form from isoprene by addition of water and than elimination of an OH-radical, in a D_2_O atmosphere, we should see a mass shift to NO^+^/70 u, and likewise for the O_2_^+^ ion. As we did not see any deuterated product ions forming when the analytes were ionized with NO^+^ and O_2_^+^ in a deuterium-oxide saturated gas stream, the hydration-dehydration mechanism indicated by the dashed lines in Figure 1 was not substantiated. Thus, the suppression of the major ion under higher humidity-conditions is not caused by a formation of the detected side products, but possibly by suppressing the ionization reaction of the analyte itself.

The SIFT-MS measurement of volatiles from several tree species mostly confirmed previous literature reports of isoprene and MBO production (Kesselmeier and Staudt, 1999). *Picea glauca, Picea abies* and *Populus nigra* were found to emit isoprene but not MBO, and *Pinus ponderosa* to emit MBO but not isoprene (Figure 5). The relative release rates we measured allow qualitative comparisons among species and individuals over the entire diurnal cycle with a frequency of 15 min per measurement. If desired, this frequency can even be increased by reducing the number of scans per measurement.

In relation to previous measurements of isoprene, the emission rates determined with SIFT-MS were typically higher than those in the literature, though still of the same magnitude (Evans et al., 1982; Steinbrecher, 1989; Janson, 1993; Kempf et al., 1996; Staudt, 1997; Niinemets et al., 2011). Together they confirm previous observations that poplar trees are higher isoprene emitters than conifers (Sharkey et al., 2008; Laothawornkitkul et al., 2009). For MBO, our measurements of *Pinus ponderosa* were lower (Supplementary Figure S5H) than in the literature (Harley et al., 1998). Differences in isoprene and MBO emission between this study and others can be explained at least in part by natural genetic variation of the trees as well as the environmental conditions of measurement. The instrument was calibrated daily using a one-point calibration with a 2 ppm VOC standard as suggested by the manufacturer. This updated the reaction time and the instrument calibration function used in Eqs. (1) and (2) and ensured stable instrument performance. To avoid systematic errors, we recommend calibrating the SIFT-MS under conditions as close to those of the intended experiment as possible. In particular, matching relative humidity is necessary if precise, quantitative values are required.

Among the individuals of *P. glauca* accession #2, one tree emitted isoprene at much higher rates than the other two trees (Supplementary Figure S5E). Genetic variation in isoprenoid formation is very commonly observed within species of *Picea* and other conifers (Martin et al., 2003; Kännaste et al., 2012). Higher isoprene emission could also originate from exposure to slightly different environmental conditions. The high isoprene-emitting tree also had a greater number of flushing buds compared to the other two trees, which could also translate into a higher isoprene emission rate.

In *P. abies*, two of the three experimental trees (entries labeled red and yellow, Table 1) emitted MBO in addition to isoprene, confirming a previous report on simultaneous emission of both volatiles (Hakola et al., 2017) from a species usually considered to be an exclusive isoprene emitter. Since the young *P. abies* trees measured were reared under controlled conditions, MBO is unlikely to have arisen from bark beetle activity. MBO and isoprene are both biosynthesized from dimethylallyl diphosphate but by different terpene synthases (Gray et al., 2011). Neither enzyme has yet been identified in *P. abies*. Since the *P. ponderosa* MBO synthase also produces a trace amount of isoprene in *in vitro* assays (Zeidler and Lichtenthaler, 2001; Gray et al., 2011), one enzyme could in principle produce both isoprene and MBO. Further work is needed on the genetic and biochemical basis of hemiterpene formation in *P. abies*.

In conclusion, we demonstrated that SIFT-MS is suitable for the simultaneous quantification of isoprene and MBO. We introduced a robust easy-to-use online method that requires minimum data treatment. In a proof of principle study, we measured the diurnal cycle of volatile emission of five different tree species with high time resolution. Single 30 cm trees were sufficient to generate robust signals. This method should be useful in applications in plant sciences, entomology, chemical ecology, and atmospheric sciences.

## Data Availability Statement

The datasets presented in this study can be found in online repositories. The names of the repository/repositories and accession number(s) can be found in the article/Supplementary Material. Code and data are published as Lehnert, A., Perreca, E., Gershenzon, J., Pohnert, G., Trumbore, S., doi: 10.17617/3.43,2020.

## Author Contributions

EP and A-SL planned and conducted the tree experiment together. EP organized the trees. A-SL conducted the standard measurements and evaluated all experimental data. EP and A-SL wrote the manuscript together. All authors assisted with data interpretation, discussion of results and helped to improve the quality of the manuscript.

## Conflict of Interest

The authors declare that the research was conducted in the absence of any commercial or financial relationships that could be construed as a potential conflict of interest.
