# Supplementary material for: Simultaneous Real-Time Measurement of Isoprene and 2-Methyl-3-Buten-2-ol Emissions From Trees Using SIFT-MS
Source: Front Plant Sci. 2020 Nov 27;11:578204. doi: 10.3389/fpls.2020.578204 (PMC7728719; doi:10.3389/fpls.2020.578204)
Supplement: Supplementary file 1 [file Data_Sheet_1.PDF]

Supplementary Material

Table S1: product ions tracked in the SIM scan

reagent ion (*m*/*z* (u))	product ion *m*/*z* (u)	ion formula (substance)
H_3O^+ (19)	19	H_3O^+
	37	$H_5O_2^+$
	55	$H_7O_3^+$
	69	$C_5H_9^+$ (isoprene / MBO)
	73	$H_9O_4^+$
NO^+ (30)	30	NO^+
	48	$NO^+ \cdot H_2O$
	68	$C_5H_8^+$ (isoprene)
	69	$C_5H_9^+$ (MBO)
O_2^+ (32)	32	O_2^+
	43	$C_3H_7^+$ (MBO)
	53	$C_4H_5^+$ (isoprene)
	58	$C_3H_6O^+$ (MBO)
	59	$C_3H_7O^+$ (MBO)
	67	$C_5H_7^+$ (isoprene)
	68	$C_5H_8^+$ (isoprene)
	69	$C_5H_9^+$ (MBO)
	71	$C_4H_7O^+$ (MBO)
	86	$C_5H_{10}O^+$ (MBO)

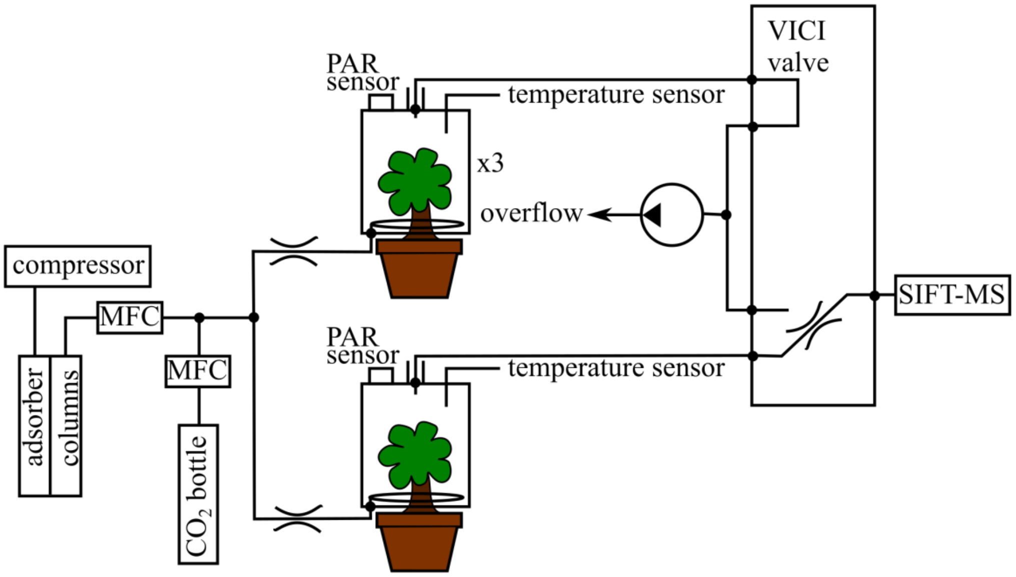

Figure S1: Scheme of the tree chamber setup

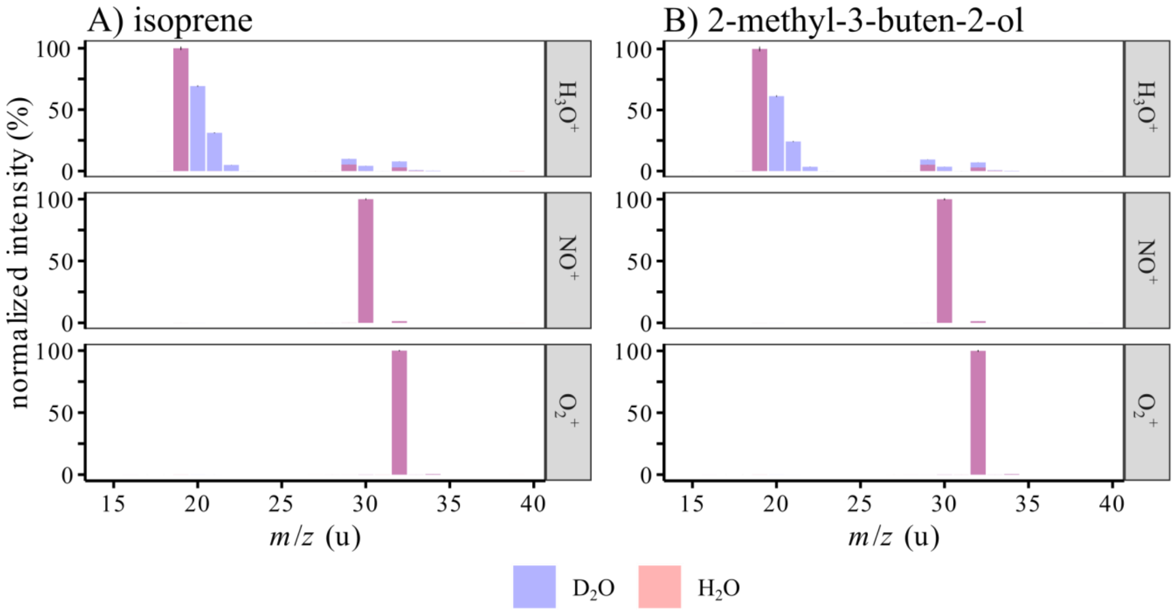

Figure S2: Relative intensity of the reagent ions (normalized to the biggest intensity in the peak area) when measuring isoprene and MBO that is humidified by normal and deuterated water.

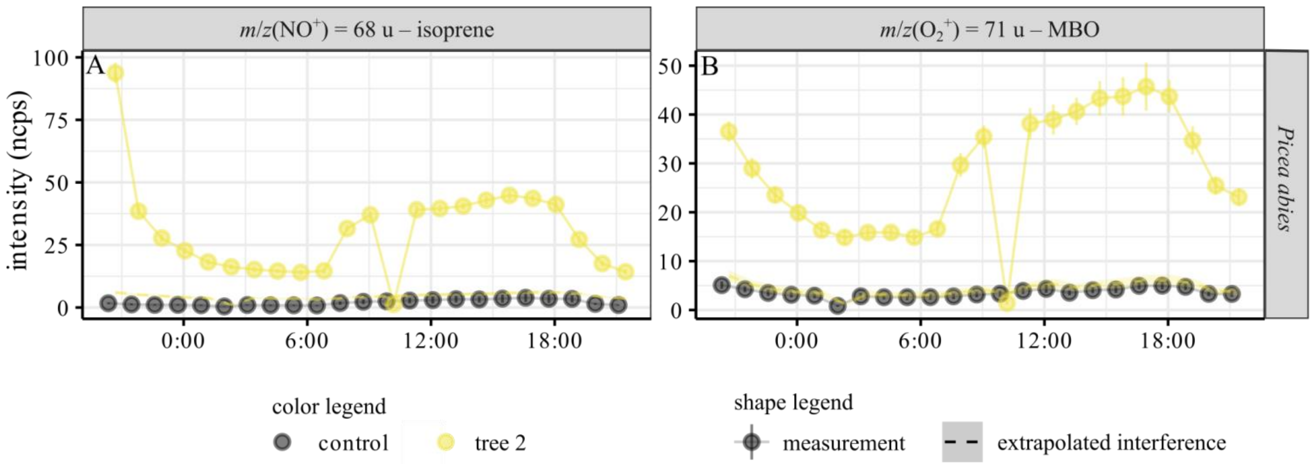

Figure S3: Diurnal cycle of isoprene and MBO emissions of the second *Picea abies* tree (for all trees, cf. Figure 5). The measured intensities were normalized to 10^6 reagent ion counts. Black: control, empty chamber. Yellow: the tree, individual 2. Dots: mean ± 95% CI of the SIFT-MS measurement. A sudden Zero value indicates instrument malfunctioning (before a firmware update, software did not always switch on the VICI valve for long measurements). Dashed lines: interference extrapolated from $m/z(NO^+)$ = 69 u (MBO interference on the isoprene signal,) and $m/z(O_2^+)$ = 67 u (isoprene interference on the MBO signal) – mean ± 95% CI. Basically, if the signal at $m/z(O_2^+)$ = 67 u is isoprene, then a maximum of 1.4% (for lower carrier gas flows) and 2.9% (for higher carrier gas flows) of this signal will be seen at $m/z(O_2^+)$ = 71 u where we measure MBO. These values are represented by the dashed lines in the graphs. If the intensity of $m/z(O_2^+)$ = 71 u is higher than this signal, we also measure MBO. This also works the other way round, for MBO interference on isoprene.

Table S2: Needle/leaf dry weights of the three replicates of the different tree species.

Tree species / replicate	1	2	3
Pinus ponderosa	7.27 g	4.40 g	2.11 g
Picea abies	22.60 g	37.00 g	14.26 g
Picea glauca sp. 1	20.34 g	28.04 g	16.05 g
Picea glauca sp. 2	9.50 g	6.04 g	5.89 g
Poplar	4.05 g	6.17 g	7.39 g

Table S3: Air flow through the chambers.

Chamber	1	2	3	Control
Flow	3.2 L/min	3.5 L/min	3.5 L/min	3.4 L/min

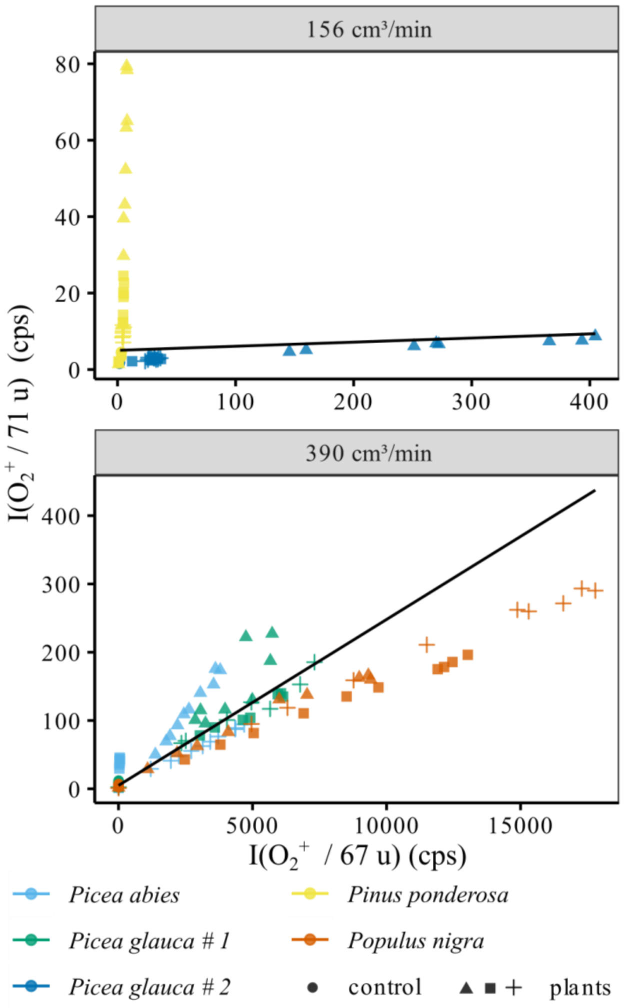

Figure S4: Ratio of the MBO signal at *m/z*(O_2^+) = 71 u versus the isoprene signal at *m/z*(O_2^+) = 67 u. The black lines mark an extrapolation of the ratio calculated based on the standard measurements under humid conditions, cf. Error! Reference source not found.**. Points below this range are considered to be the result of isoprene interference on the MBO signal, points above the range are considered to represent genuine MBO signals.**

Figure S5: Isoprene and MBO emission rate in µg per g leaf/needle dry weight per hour (mean ± 95% CI) for the five different tree species evaluated. The extrapolated interference background was subtracted before calculating the emission rates.

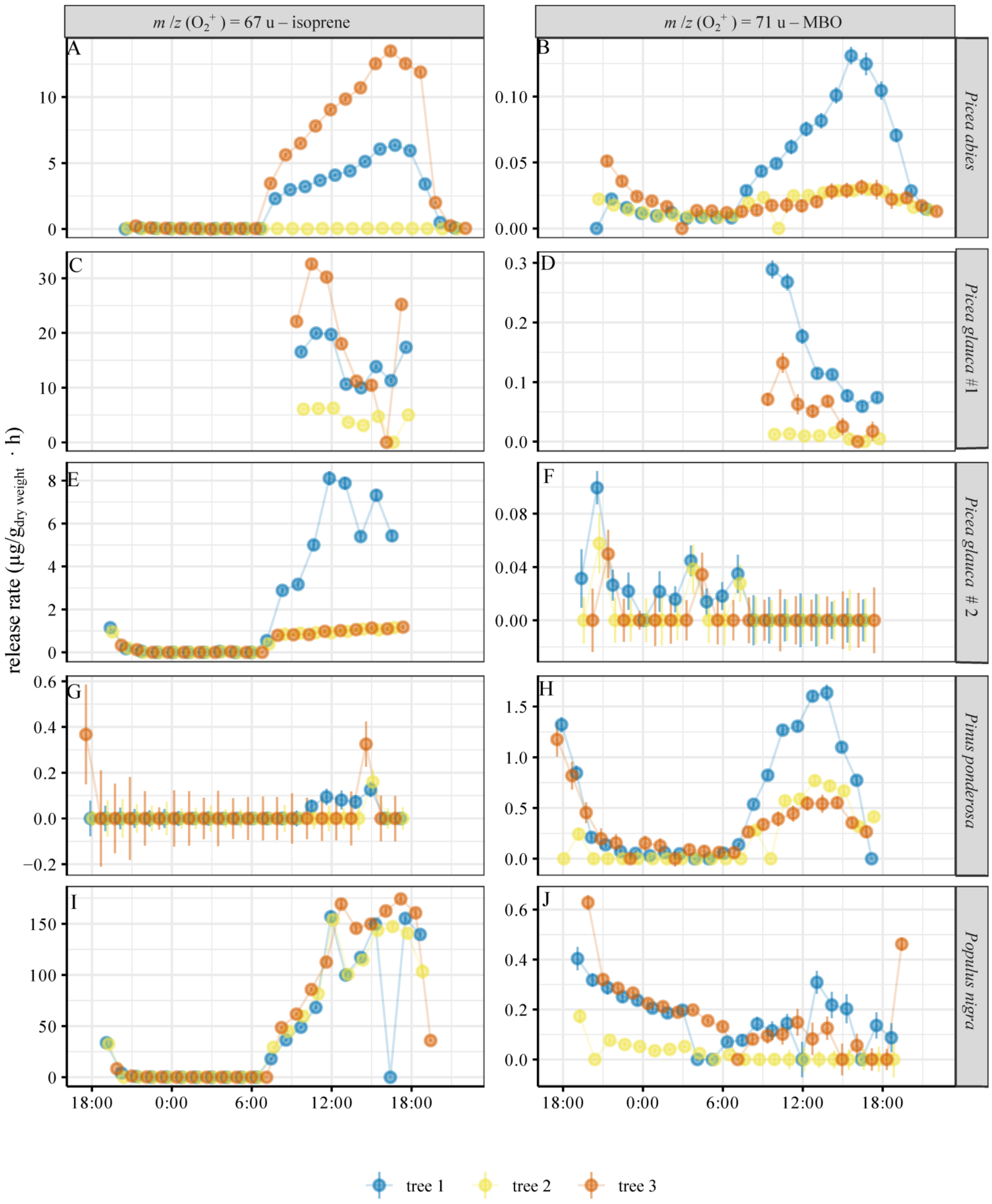

Figure S6: Isoprene and MBO emission rate in µg per g leaf/needle dry weight per hour (mean ± 95% CI) for the five different tree species evaluated. Both are based on their signals upon reaction with O_2^+. The extrapolated interference background was subtracted before calculating the emission rates.

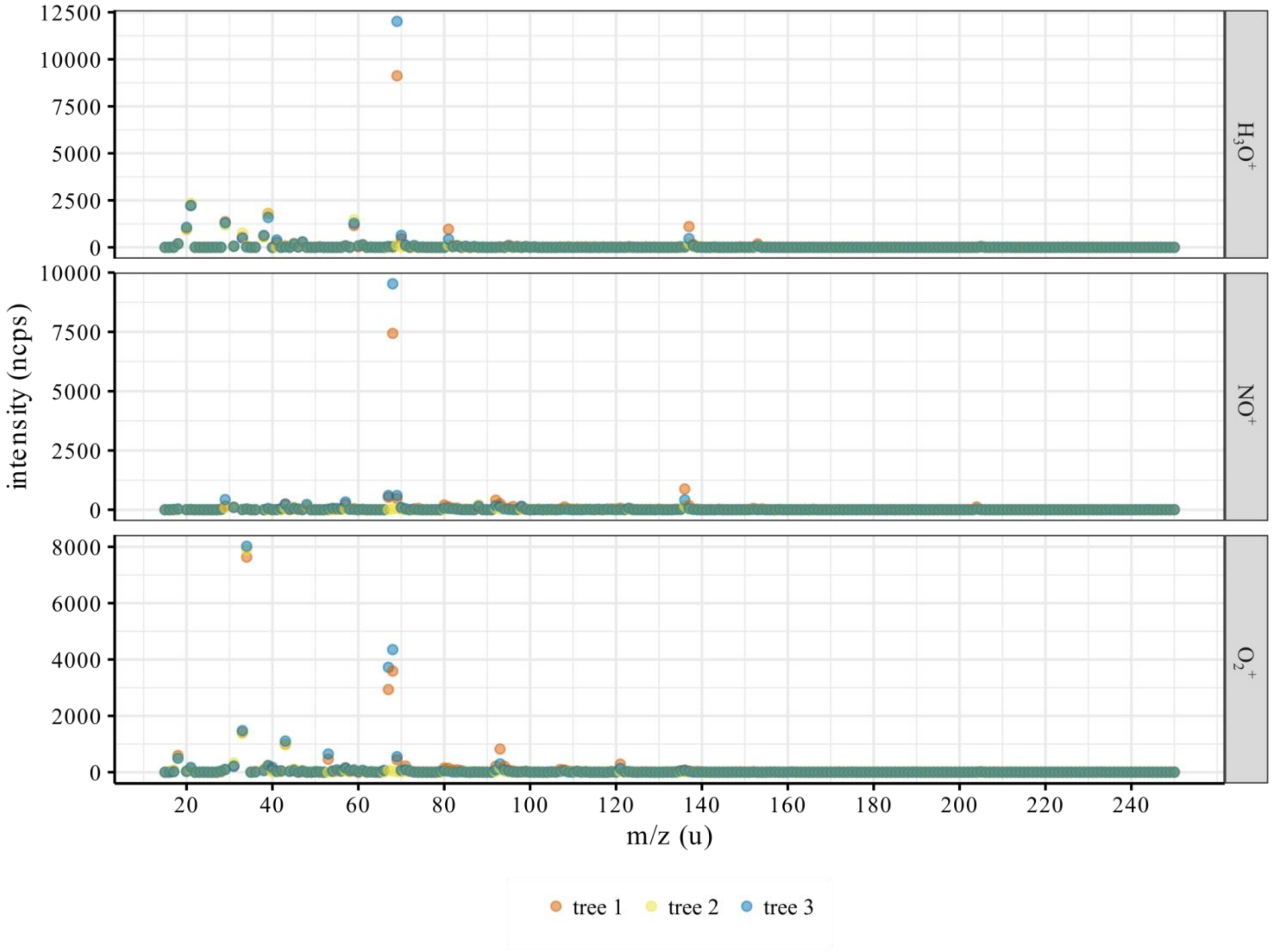

Figure S7: SIFT-MS mass spectra of the three *P. abies* trees at 3 pm (where the emissions are highest in this tree). The intensities are normalized to 10^6 reagent ion counts. Since only one spectrum was measured, no error bars could be calculated. *m/z* = 19, 30, 32, 37 u (the reagent ions) are not shown due to their high intensity. Monoterpenes as sum parameter can be measured on *m/z*(H_3O^+) = 137 u, *m/z*(NO^+) = 136 u, and *m/z*(O_2^+) = 136 u, sesquiterpenes on *m/z*(H_3O^+) = 205 u, *m/z*(NO^+) = 204 u, and *m/z*(O_2^+) = 204 u.
